# Prognostic potential of circulatory miR-19a-3p, miR-19b-3p, and miR-329–3p for future hypertension diagnosis

**DOI:** 10.1097/HJH.0000000000004272

**Published:** 2026-03-05

**Authors:** Daria Kostiniuk, Antti Blankenstein, Sonja Rajić, Joanna Ciantar, Nina Mononen, Leo-Pekka Lyytikäinen, Ilkka Seppälä, Pashupati P. Mishra, Markus Juonala, Melanie Waldenberger, Marko Elovainio, Niku Oksala, Mika Kähönen, Nina Hutri, Olli Raitakari, Marcus E. Kleber, Winfried März, Terho Lehtimäki, Saara Marttila, Emma Raitoharju

**Affiliations:** a Molecular Epidemiology Group; b Department of Clinical Chemistry, Faculty of Medicine and Health Technology, Tampere University; c Fimlab Laboratories; d Finnish Cardiovascular Research Centre Tampere, Faculty of Medicine and Health Technology, Tampere University, Tampere; e Division of Medicine Turku University Hospital and Department of Medicine, University of Turku, Turku, Finland; f Research Unit Molecular Epidemiology, Institute of Epidemiology, Helmholtz Zentrum, German Research Centre for Environmental Health, Berlin, Germany; g Department of Psychology, Faculty of Medicine, University of Helsinki, Helsinki; h Centre for Vascular Surgery and Interventional Radiology, Wellbeing Services County of Pirkanmaa, Tampere University Hospital; i Department of Clinical Physiology, Tampere University Hospital, and Faculty of Medicine and Health Technology, Tampere University; j Department of Pediatrics, University of Tampere and Tampere University Hospital, Tampere; k Centre for Population Health Research, University of Turku and Turku University Hospital; l Research Centre for Applied and Preventive Cardiovascular Medicine, University of Turku; m Department of Clinical Physiology and Nuclear Medicine, University of Turku and Turku University Hospital; n InFLAMES Research Flagship, University of Turku, Turku, Finland; o Vth Department of Medicine (Nephrology, Hypertensiology, Endocrinology, Diabetology, Rheumatology), Medical Faculty of Mannheim, University of Heidelberg, Heidelberg; p SYNLAB MVZ Humangenetik Mannheim, Mannheim; q Synlab Academy, SYNLAB Holding Deutschland GmbH, Augsburg & Mannheim, Germany; r Tampere University Hospital, Wellbeing Services County of Pirkanmaa; s Gerontology Research Centre, Tampere University, Tampere, Finland

**Keywords:** blood pressure, essential hypertension, miRNA, prospective study

## Abstract

MicroRNAs have been suggested as essential hypertension biomarkers, but evidence remains inconclusive due to limited high-throughput studies in population cohorts.

We analyzed data from the Young Finns Study (YFS) from 2011 and 2018–2020 to assess cross-sectional and prospective associations between circulatory microRNAs, blood pressure (BP), and hypertension. Hypertension risk prediction potential was assessed using nested logistic and Weibull survival models; model performance was evaluated with likelihood ratio (LR) test and c-statistic. All models were adjusted with relevant risk factors.

In 2011, whole blood microRNAs were profiled for 871 individuals (83 with hypertension); in 2018–2020, 760 were re-examined, with 67 newly diagnosed. Cross-sectionally, 16 miRNAs correlated with BP (Spearman, *P*_FDR_ < 0.05); miR-122–5p (fold change = 1.33) and miR-144–5p (fold change = -1.10) differentiated hypertensive individuals (*U* test, *P*_FDR_ < 0.05). Associations persisted in adjusted regression models and some replicated in LURIC (*n* = 999) and YFS serum data (*n* = 126). Prospectively, miR-19a-3p [odds ratio (OR) = 1.51, 95% confidence interval (95% CI): 1.14–2.18], miR-19b-3p (OR = 1.50, 95% CI:1.11–2.04), and miR-329–3p (OR = 0.58, 95% CI: 0.39–0.74) levels prognosed hypertension incident. miR-329–3p improved model fit (LR test, *P* = 2.85×10^–4^) and discrimination (c-statistic = 0.849, Δ = 0.026). miR-19b-3p predicted time to onset (hazard ratio = 2.13, 95% CI: 1.38–4.45), improving model fit (LR test, *P* = 0.0012) and time-dependent discrimination at 7 and 8-year horizons.

Our findings highlight both novel and previously reported miRNAs associating with BP and hypertension and suggest that miR-329–3p, miR-19a-3p, and miR-19b-3p as promising candidates for further investigation in hypertension risk prediction.

## INTRODUCTION

Essential hypertension is a condition characterized by persistently high blood pressure with no identifiable secondary cause, affecting over a billion people globally [1]. Despite its concerning prevalence, only 31–77% of people with hypertension are aware of their condition, and just 9–43% have it medically controlled [2]. Thus, hypertension remains a leading, yet often preventable, risk factor for cardiovascular and renal diseases, contributing to approximately 8.5 million deaths annually [2,3].

Hypertension is a multifactorial disease, caused by a combination of environmental factors, aging, and genetic predisposition. Its pathophysiology is heterogenous as it involves a complex interaction between neural, renal, and humoral systems regulating blood pressure (BP) [4]. This complexity is underscored in the variable therapeutic response to antihypertensive agents, with the majority of hypertension patients (about 70%) requiring combination therapy to achieve BP control [5,6]. Our understanding of hypertension development remains incomplete and one potential avenue for its improvement, as well as early diagnosis and treatment of hypertension, is the identification and use of biomarkers.

MicroRNAs (miRNAs) are short, approximately 22 nucleotides long, single-stranded noncoding RNAs, functioning as regulators of gene expression at the posttranscriptional level [7]. It has been extrapolated that the human genome encodes for 2300 miRNAs [8]. They exhibit distinct profiles across various cell types and tissues and mediate cell-to-cell communication when released into circulation [9]. In pathological conditions, circulatory miRNA profiles undergo substantial change [10–12], which makes them potential biomarker candidates with biological relevance and convenient physicochemical properties [13,14].

MicroRNAs target genes involved in BP regulation, including those associated with sympathetic and endothelial signaling, oxidative stress, and hypertension risk genes [15]. Experimental evidence shows that transferring exosomes from hypertensive to normotensive rats raises BP and promotes subsequent vascular pro-atherogenic changes [16]. Targeted associational studies also connect specific circulatory miRNAs involved in vascular remodeling, endothelial dysfunction, and atherosclerosis development with hypertension [17], particularly miR**-**21 [18], miR-30 family [19–21], miR**-**34a [22,23], miR**-**92a [24–26], miR-126 [20,22,27], miR-143/145 cluster [27,28], and let-7 family [29–32].

However, it is unclear whether miRNAs could serve as reliable biomarkers for hypertension, as findings from associational studies evaluating the association between the circulatory miRNAs and hypertension often yield inconsistent findings. These inconsistencies are likely due to variations in blood sample types, small sample sizes (*n* < 100), differences between focused and unbiased miRNA profiling, as well as variations in miRNA profiling methodologies [17]. Additionally, most documented associations between circulatory miRNAs and hypertension have been identified through cross-sectional studies in patient-based cohorts, which limits their ability to establish miRNA relevance in the early stages of the disease and the generalizability of the findings.

In the present study, we utilized the longitudinal Young Finns Study (YFS) cohort to explore miRNAs associated with BP and hypertension in cross-sectional and prospective settings. Our primary focus was to identify miRNAs with the potential to enhance the hypertension prediction value beyond traditional risk factors and the well validated Framingham Heart Study (FHS) hypertension risk model [33–35].

## MATERIALS AND METHODS

### Study cohorts

The Young Finns Study (YFS) [36] is an ongoing prospective follow-up study intended to study cardiovascular disease (CVD) risk determinants originating in childhood and youth. The baseline study, conducted in 1980, included six age groups, totaling 3596 participants aged three to 18 years. Starting from 1980, the individuals were followed up at three-year intervals until 1992, then again in 2001, 2007, 2011, and 2018–2020. The data utilized in this study originates from the 2011 and 2018–2020 follow-ups. In 2011, participants were aged 37–49 years, with 50% being women.

MicroRNA profiles were analyzed from 871 randomly selected whole blood samples collected during the 2011 follow-up. This miRNA data was used to investigate both cross-sectional associations between HT and BP, and prospective associations with HT from the 2018–2020 follow-up. Of the individuals with 2011 miRNA profiling, 760 participated in 2018–2020 follow-up (12.7% drop out rate). Additionally, among 145 individuals, serum miRNA profiling was conducted in addition to whole blood profiling. The serum miRNA data served as replication for the cross-sectional whole blood data-based findings.

The Ludwigshafen Risk and Cardiovascular Health (LURIC) [37] is a patient cohort investigation that includes individuals with and without CVD at its baseline, comprising of individuals who were hospitalized between June 1997 and May 2001 for coronary artery disease (CAD) investigation. In our study, we are focusing on subset of LURIC participants (*n* = 999) randomly selected for plasma miRNA profiling. The subset age range spans from 49 to 71 years, with men constituting 69% of the group. We used the LURIC phenotypic and miRNA profiling data to replicate cross-sectional YFS-based findings.

### Ethical statements

YFS and LURIC data have been collected following the Helsinki Declaration requiring written informed consent from the study participants. The YFS was approved by the first ethical committee of the Hospital District of Southwest Finland and by local ethical committees [36]. The LURIC study was approved by the ethics review committee at the Landesärztekammer Rheinland-Pfalz in Mainz, Germany (LURIC,#83725597 (1394)) [37].

### Hypertension diagnosis, blood pressure, and covariate measurements

BP measurements for YFS and LURIC participants were obtained in-office using automatic BP monitors. In YFS, BP was measured in a sitting position with 5-min intervals between each of the three readings, and the average SBP and DBPs were used for the analysis. In LURIC, the average of five BP readings measured at least 30 s apart was used in the analysis. According to the European Society of Cardiology guidelines [38], participants were classified as hypertensive if their SBP was greater than 140 mmHg or DBP greater than 90 mmHg. Participants were considered as having hypertension diagnosis if they self-reported being diagnosed with the condition or were prescribed corresponding medication.

For the statistical analyses, comprehensive covariate data from the YFS and LURIC were utilized. These included sex, age, BMI and enzymatically measured serum total cholesterol (TC), and alanine aminotransferase (ALT). Covariates also encompassed steatotic liver disease measured via ultrasound, type 2 diabetes (T2D), as well as questionnaire data on alcohol consumption, smoking habits, work-related stress, and parental hypertension. Details on covariate measurements are provided in Supplementary methods.

### Whole blood and serum miRNA profiling in Young Finns Study

In the YFS, RNA isolation and quality control for whole blood and serum were performed as described previously [39,40]. Both whole blood and serum samples from the 2011 follow-up underwent miRNA expression profiling with the TaqMan**™** OpenArray**™** MicroRNA Panel (Life Technologies) containing 758 miRNA probes. The panel's functionality was validated previously [41].

For the whole blood miRNA expression data, primary analysis was performed with Expression Suite Software version 1.0.1, accepting assays with Amplification score more than 1 and Cq Confidence more than 0.7. Profiling was successful on 871 whole blood samples for 243 miRNAs. Batch effects were corrected by adjusting the data with the first ten principal components. Serum data QC followed the same criteria, with successful profiling of 145 serum samples and 160 miRNA species. Batch correction was done with ComBat [42].

Fold change of miRNA expression was determined using the ΔΔCt method. In whole blood, change in threshold cycle (ΔCt) was calculated by subtracting the Ct values of reference genes (RNU6, RNU44, and RNU48), while serum data underwent global mean normalization and between-sample normalization using the quantile normalization method in R [43].

### Plasma miRNA expression profiling in LURIC

In LURIC, plasma isolation was conducted within 15–20 min postsample collection, utilizing centrifugation and citrate as an anticoagulant treatment. MicroRNA isolation, quality control, and expression profiling were performed by Hummingbird Diagnostic (formerly CBC) using an Agilent microarray (Agilent Technologies) containing 2539 miRNA probes. The analysis followed standard protocol and data preprocessing was done with AgiMicroRna Bioconductor packages in R. Plasma miRNA expression data was quantile normalized, and 269 miRNAs expressed in at least 75% of the samples were retained for further analysis. The remaining miRNA expression values underwent log2 transformation. After quality control filtering, profiling was successful 999 plasma samples.

### Genome-wide expression analysis

In YFS, the gene expression levels from 2011 YFS follow-up whole blood samples were analyzed with the Illumina HumanHT-12 version 4 Expression BeadChip (Illumina Inc.). Raw illumina probe data was exported from Beadstudio and processed with R. Data processing is described in more detail elsewhere [44]. The expression analysis was successful in 743 of the 871 samples with miRNA expression profiles.

### Statistical analysis

#### Cross-sectional analysis

**FIGURE 1:**
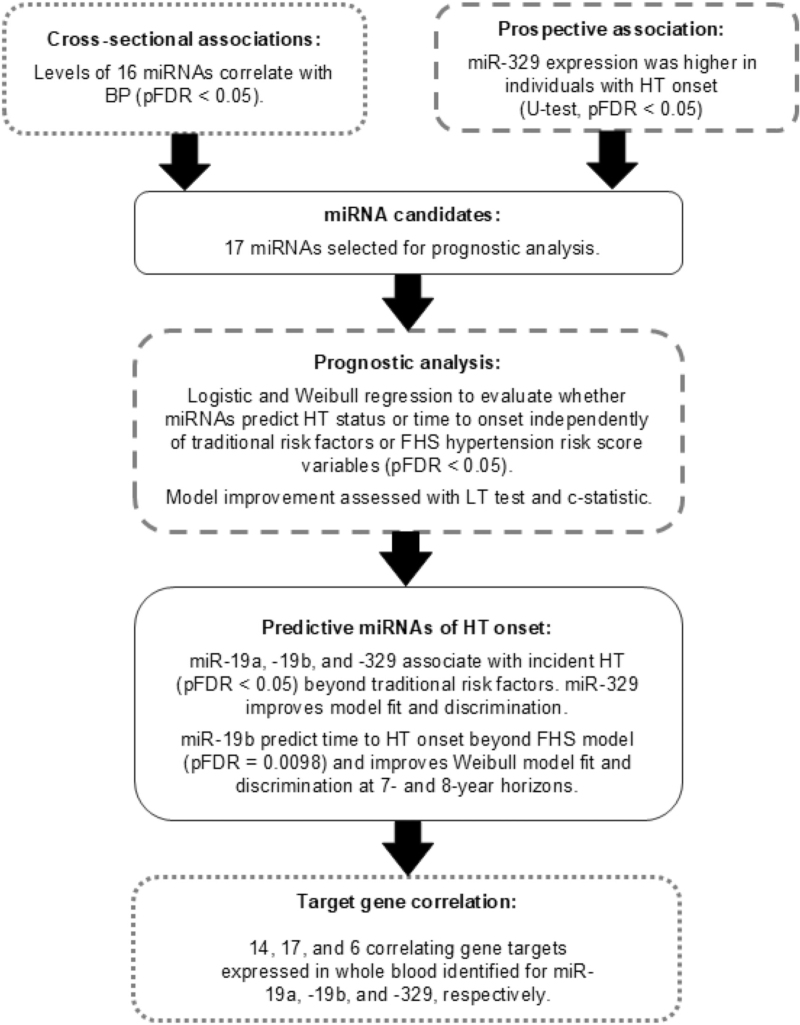
Study flow chart. Boxes with dotted outline represent cross-sectional analyses and ones with dashed outline represent prospective analysis.

Flow chart of statistical analysis is depicted in Fig. 1. In whole blood 2011 YFS data, miRNAs significantly correlating with BP underwent further assessment using two linear regression models with dependent variables being SBP or DBP. Model 1 was adjusted for age, sex, and BMI, while fully adjusted Model 2 additionally included smoking status, alcohol usage, ultrasound-defined steatotic liver disease, work-related stress, T2D, and TC. Differences in miRNA profiles between hypertensive and normotensive individuals, and those diagnosed and nondiagnosed with hypertension, were assessed using the two-tailed Mann-Whitney *U* test. Significant miRNAs were further evaluated for their association with hypertension with two logistic regression models, adjusted with the same covariates as the linear regression models. To account for the use of BP-lowering medication, 15 and 10 mmHg were added to SBP and DBP values, respectively [45].

BP and hypertension associations in whole blood were re-evaluated separately for men, women, and normotensive individuals using the same methodologies. To replicate the findings from the YFS 2011 whole blood data, the same analyses were also performed for YFS 2011 serum and LURIC plasma miRNA data. The linear and logistic regression models were adjusted with similar covariates.

All statistical tests were done in R [46]. False discovery rate (FDR) correction was applied for Mann-Whitney *U*-tests and Spearman correlations conducted on whole blood miRNA data, with *P*_FDR_ less than 0.05 considered significant. In the replication and stratified analysis, the Mann-Whitney *U*-tests and Spearman correlations were considered significant if the nominal *P* value was less than 0.05. In linear and logistic models, *P* value less than 0.05 was considered significant. The reported β coefficients are standardized. To ensure data comparability and normality, miRNA profiles were reverse normalized, and nonnormal regression model covariates were log-transformed. Linear and logistic regression analyses were assessed for the assumption of residual or logit normality.

#### Prospective analysis

We evaluated the ability of miRNAs to predict HT diagnosed during period between the 2011 and 2018–2020 YFS follow-up among participants without HT diagnosis at baseline (2011). Candidate miRNAs for risk prediction analysis were those that cross-sectionally associated with BP or hypertension diagnosis at baseline (significant correlation for at least one BP parameter, *P*_FDR_ < 0.05) and differentiated individuals with and without hypertension in 2018–2020 according to a Mann-Whitney *U*-test (*P*_FDR_ < 0.05).

Risk prediction analyses were conducted using logistic and Weibull regression in nested models, where two models with and without inclusion of the candidate miRNA were compared (miRNA model vs. base model). For logistic regression the base model included age, sex, BMI, T2D, TC, steatotic liver disease, work-related stress, tobacco and alcohol usage, as well as mean arterial pressure (MAP, 1/3×SBP+2/3×DBP). Weibull survival model was based on FHS HT risk prediction model that was refitted to YFS data and included sex, BMI, smoking, SBP and DBP, parental hypertension, and interaction term between DBP and age [34].

A miRNA was considered a significant risk predictor if it remained significantly associated with HT onset or time to onset after adjustment for covariates in the base model (*P*_FDR_ < 0.05). Odds ratios (ORs), hazard ratios, and their 95% confidence interval (95% CI) were estimated using 1000 bootstrap replicates. Model fit improvement was assessed by LR test comparing nested models. Discrimination for logistic regression models was assessed using the area under receiver operating characteristics curve (AUROC) with *pROC* package [47]. For Weibull regression, time-dependent c-statistics were evaluated for 7 and 8-year horizons with *timeROC* package [48]. 95% CIs for the AUROCs were computed with 1000 stratified bootstrap replicates.

### MicroRNA correlation with validated targets

Validated gene targets for miRNAs were sourced from the 2022 release of miRWalk [49]. Available targets from the YFS 2011 follow-up genome-wide expression data were correlated using Spearman rank method. Both positive and negative correlations were accepted, and gene targets were considered significant if they exhibited correlation with respective miRNA levels at a *P*_FDR_ < 0.05.

## RESULTS

### Demographics: Young Finns Study

The 2011 YFS follow-up with whole blood miRNA profiling included individuals aged 37–49 years, with 50% being women. Among them, 83 were diagnosed with hypertension, with a higher prevalence in men (12.1%) compared to women (8.1%). Participants with hypertension exhibited higher BP than those without the diagnosis. However, 21% of those without hypertension were hypertensive (SBP > 140 mmHg or DBP > 90 mmHg). Individuals with hypertension had worse metabolic health than the rest of the group, as summarized in Table S1. Demographics for the smaller serum miRNA profiling group (*n* = 145) are summarized in Table S2.

In the subsequent YFS 2018–2020 follow-up, 760 individuals from the 2011 whole blood subset participated. Among them, 67 had hypertension diagnosis in 2011, and 78 were newly diagnosed during the period between follow-ups (mean duration 7.1 ± 0.43 years). Already in the 2011 follow-up, individuals who were later diagnosed with HT were older and displayed worse metabolic health compared to those without hypertension, with nearly 60% being hypertensive. By the 2018–2020 follow-up, the overall population showed a trend of worsening metabolic health, with those newly diagnosed with hypertension continuing to differ from those without hypertension (Table S3).

### Demographics: LURIC

The baseline subset of LURIC participants subjected to miRNA profiling comprised a total of 999 individuals. The ages of participants ranged from 49 to 71 years, and the majority were men (69%). HT diagnosis was highly prevalent, with 90% of the subset being diagnosed with hypertension. Demographics for the LURIC miRNA profiling subset are summarized in Table S4.

### Whole blood miRNAs associate with blood pressure in cross-sectional setting

As summarized in Table 1 and detailed in Tables S5 and S6, four miRNAs significantly correlated with both SBP and DBP, while 11 miRNAs showed correlations specifically with either SBP or DBP. All four miRNAs correlating with both BP parameters were significantly associated with the SBP and DBP in regression Model 1 (adjusted for sex, age, and BMI). In the fully adjusted regression Model 2 (adjusted for sex, age, BMI, smoking status, alcohol usage, steatotic liver disease, work-related stress, T2D, and TC), miR-122–5p and -200c-3p were significantly associated with both SBP and DBP, while miR-183–3p was associated with SBP and miR-185–5p with DBP.

**TABLE 1:** Summary of whole blood miRNA associations with blood pressure and replication in serum and plasma

	YFS 2011 WHOLE BLOOD					YFS 2011 SERUM					LURIC PLASMA				
			
		SBP		DBP			SBP		DBP			SBP		DBP	
									
	*n*	*r/*β	*P*	*r/*β	*P*	*n*	*r/*β	*P*	*r/*β	*P*	*n*	*r/*β	*P*	*r/*β	*P*
miR-122-5p															
Correlation	700	0.21	7.38×10^–6*^	0.22	1.75×10^–6*^	145	0.32	0.0057	0.36	5.70×10^–4^	Not significant				
Model 1	698	0.08	0.018	0.11	8.00×10^–4^	144	0.08	0.327	0.17	0.029					
Model 2	597	0.07	0.029	0.08	0.025	132	0.06	0.433	0.14	0.073					
miR-885–5p															
Correlation	865	0.11	0.022*	0.09	0.098*	145	0.38	2.51×10^–4^	0.41	2.74×10^–5^	NA				
Model 1	862	0.02	0.534	0.04	0.177	144	0.11	0.154	0.22	0.004					
Model 2	735	0	0.901	0.03	0.293	132	0.08	0.308	0.17	0.032					
miR-18a-3p															
Correlation	861	-0.07	0.359*	-0.11	0.041*	142	-0.13	0.13	-0.18	0.03	NA				
miR-185–5p															
Correlation	868	-0.15	7.75×10^–4*^	-0.17	4.38×10^–5*^	Not significant					999	-0.1	9.17×10^–4^	-0.1	0.0039
Model 1	865	-0.08	0.0041	-0.1	3.48×10^–4^						999	-0	0.193	-0.1	0.082
Model 2	738	-0.05	0.079	-0.07	0.014						999	-0	0.175	-0.1	0.069
miR-19b-3p															
Correlation	864	-0.12	0.021*	-0.10	0.061*	Not significant					999	-0.1	0.0042	-0.1	0.0012
Model 1	861	-0.08	0.0047	-0.05	0.087						999	-0.1	0.076	-0.1	0.016
Model 2	734	-0.05	0.082	-0.02	0.432						999	-0.1	0.111	-0.1	0.022
miR-223–3p															
Correlation	861	-0.12	0.021*	-0.04	0.596*	Not significant					999	-0.1	0.0052	-0.1	0.0065
miR-423–5p															
Correlation	811	-0.08	0.275*	-0.12	0.017*	Not significant					999	-0	0.66	-0.1	0.029
miR-150–5p															
Correlation	859	-0.12	0.017*	-0.07	0.319*	Not significant					Not significant				
Model 1	856	-0.05	0.157	-0.09	0.843										
Model 2	729	-0.06	0.027	-0.03	0.382										
miR-144–5p															
Correlation	860	-0.10	0.089*	-0.14	0.0029*	Not significant					NA				
Model 1	857	-0.05	0.076	-0.09	0.0014										
Model 2	731	-0.07	0.018	-0.1	6.37×10^–4^										
miR-142–3p															
Correlation	863	-0.10	0.089*	-0.13	0.0057*	Not significant					NA				
Model 1	860	-0.02	0.506	-0.07	0.025										
Model 2	734	-0.01	0.72	-0.04	0.13										
miR-19a-3p															
Correlation	851	-0.10	0.089*	-0.11	0.029*	Not significant					NA				
miR-363–3p															
Correlation	854	0.10	0.089*	0.13	0.0043*	NA					NA				
Model 1	851	0.05	0.083	0.1	8.76×10^–4^										
Model 2	729	0.03	0.289	0.07	0.014										
miR-183–3p															
Correlation	852	0.15	0.0017*	0.14	0.0029*	NA					NA				
Model 1	849	0.08	0.004	0.08	0.0068										
Model 2	723	0.06	0.039	0.05	0.065										
miR-200c-3p															
Correlation	776	-0.12	0.022*	-0.11	0.041*	NA					NA				
Model 1	774	-0.07	0.015	-0.07	0.019										
Model 2	664	-0.09	0.004	-0.09	0.0028										
miR-20b															
Correlation	849	-0.06	0.42*	-0.11	0.041*	NA					NA				
Model 1	846	-0.04	0.172	-0.07	0.015										
Model 2	720	-0.03	0.355	-0.07	0.015										

“Not significant” indicates no significant association between a miRNA and BP in correlation or linear regression analysis, while “NA” indicates that a miRNA is not available from the respective data. Correlation *r* is obtained from Spearman rank correlation. Model 1 was adjusted for age, sex, and BMI. Model 2 is adjusted for age, sex, BMI, smoking status, alcohol usage, ultrasound-identifiable steatotic liver disease, type 2 diabetes, total cholesterol, and blood pressure medication.

Table summarizes whole blood miRNAs that correlated with blood pressure after FDR-multiple testing correction, along with the replication of these associations in YFS 2011 serum and LURIC plasma miRNA data. - FDR-corrected *P*-values are indicated with an asterisk (^*^). Linear regression analysis and replication correlation analyses are considered significant if *P* < 0.05. Only results from correlation analysis are shown if regression analysis did not yield significant results. The full result tables for correlation and regression analysis, including 95% confidence intervals for *r* and standardized β coefficients, are provided in Tables S5 and S6, respectively.

Among the four miRNAs correlating with only SBP, miR-19b-3p was significantly associated with SBP in Model 1, while miR-150–5p with DBP in Model 2. Among the seven miRNAs correlating with DBP alone, miR-142–3p associated with DBP in Model 1, while miR-20b, miR-144–5p, and miR-363–3p remained significantly associated with DBP in both models.

Most miRNAs that associated with BP parameters in all individuals also demonstrated associations with BP in the subset of normotensive individuals (Table S7). In the sex-stratified analysis, miR-122–5p, and miR-185–5p correlated with both SBP and DBP in both men and women, with the association between these miRNAs and BP remaining significant only for women in Model 1 and in the fully adjusted Model 2. Levels of miR-144–5p correlated significantly with DBP and were associated with DBP in both men and women in Model 1 and in the fully adjusted regression Model 2 (Table S8).

### Distinguishing whole blood miRNA profiles in individuals with and without hypertension diagnosis, and between normotensive and hypertensive groups

In the whole blood data, none of the profiled miRNAs could differentiate individuals with hypertension diagnosis. However, when comparing normotensive and hypertensive individuals (>140/90 mmHg), miR-122–5p and miR-144–5p exhibited differential expression in hypertensive individuals (Mann-Whitney *U*-test, FC = 1.27, *P*_FDR_ = 0.0012, FC = -1.08, p_FDR_ = 0.035, respectively). In logistic regression models, miR-122–5p was significantly associated with hypertensive phenotype in Model 1 (OR = 1.25, *P* = 0.027), while miR-144–5p was significantly associated with hypertensive phenotype in both Model 1 (OR = 0.72, *P* = 6.65×10^–4^) and fully adjusted Model 2 (OR = 0.66, *P* = 1.33×10^–4^).

### Replication of the cross-sectional whole blood Young Finns Study findings with serum and plasma miRNA profiles

The YFS 2011 serum and LURIC plasma analysis aimed to replicate the associations between miRNAs and BP observed in whole blood (Table 1). The full results of the replication are represented in Tables S5 and S6. None of the miRNAs associated with BP in whole blood, replicated in both serum and LURIC plasma. In YFS 2011 serum, miR-122–5p and miR-885–5p correlated positively with both SBP and DBP, replicating the whole blood findings. However, in Model 1 and the fully adjusted Model 2, miR-122–5p lost significance while miR-885–5p retained significance for DBP, which contrasts with the whole blood data.

The negative correlation observed between miR-185–5p, -19b-3p, -223–3p, and -423–5p and BP in whole blood was replicated by LURIC plasma. While in the whole blood data, miR-19b-3p was associated with only DBP, in LURIC miR-19b-3p correlated with both BP parameters, with a significant association with DBP in both Model 1 and the fully adjusted Model 2. In LURIC, no miRNA differed between normotensive and hypertensive individuals.

### Changes in miRNA profiles before the hypertension diagnosis

We utilized whole blood miRNA profiling data from the YFS 2011 follow-up to investigate prospective differences in miRNA profiles between individuals who were newly diagnosed with hypertension by the YFS 2018–2020 follow-up and those who remained without hypertension diagnosis. Among the 243 profiled whole blood miRNAs, miR-329–3p was differentially expressed between these two groups, with individuals who were newly diagnosed with hypertension exhibiting lower miR-329–3p levels, as determined by the Mann-Whitney *U* test with multiple testing correction (FC = -1.24, *P*_FDR_ = 0.046).

### Risk prediction potential of circulatory miRNA profiles for hypertension diagnosis in a 6-to-9-year period

We assessed the risk prediction potential of candidate miRNAs using logistic regression and Weibull regression, evaluating their association with hypertension status at follow-up and the time to hypertension onset, accounting for interval-censored follow-up data, respectively. The candidate miRNAs for prospective analysis were selected based on their cross-sectional association with BP or hypertension in the cross-sectional analysis of the YFS whole blood data (Table 1) and miR-329–3p, the profiles of which differed between those who became diagnosed with hypertension and those who did not.

**FIGURE 2:**
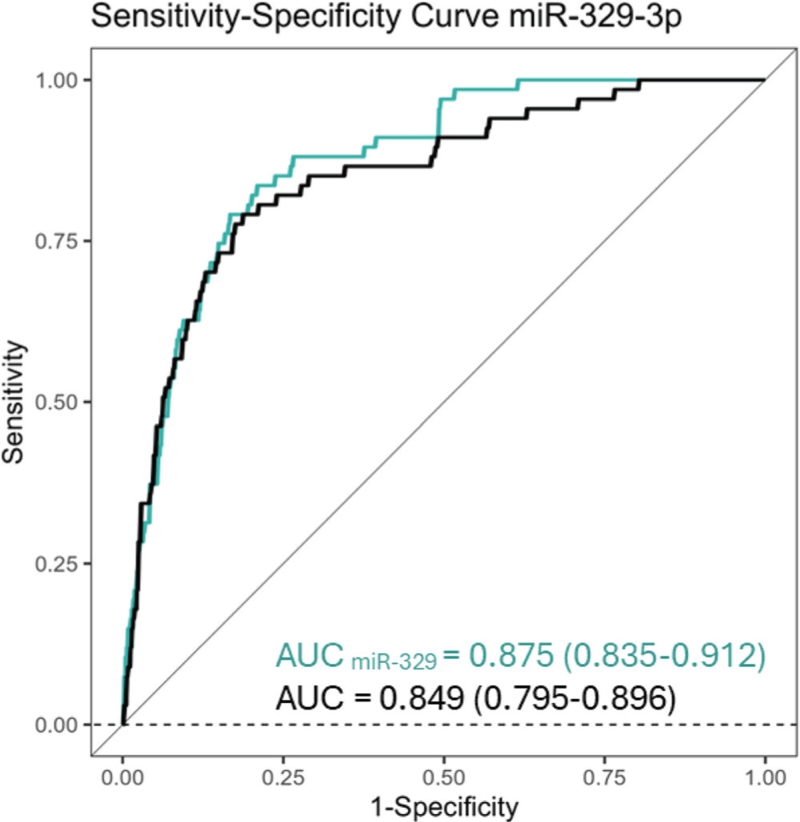
Receiver operation characteristics curves comparing logistic regression models with and without miRNAs.

Of these 16 candidate miRNAs, miR-19a-3p, -19b-3p, and -329–3p associated with diagnosed hypertension in the period between follow-ups after multiple testing correction (*P*_FDR_ < 0.05, Table S9) and adjustment for age, sex, MAP, TC, steatotic liver disease, T2D, stress, tobacco and alcohol usage (Table 2). Inclusion of these miRNAs in the base model significantly improved its fit (LR test *P* < 0.05, Table 2). However, only miR-329–3p improved the risk prediction performance also by increasing AUC (Fig. 2). Both the association of miR-329–3p with hypertension incidence and the improvement in AUROC were also observed in sex-stratified analyses.

**TABLE 2:** Model fit and discrimination improvement after miRNA incorporation to the risk prediction model

	*n*	OR (CI 95%)	*P*	AUC_miRNA_ (CI 95%)	AUC_base_ (CI 95%)	pLR
miR-329–3p						
All	565	0.58 (0.39–0.74)	5.25×10^–4^	0.875 (0.835–0.912)	0.849 (0.795–0.896)	3.85×10^–4^
Men	233	0.49 (0.22–0.89)	0.0077	0.898 (0.824–0.950)	0.873 (0.792–0.940)	0.0051
Women	332	0.62 (0.37–0.88)	0.021	0.865 (0.809–0.916)	0.844 (0.776–0.905)	0.021
miR-19a-3p						
All	589	1.51 (1.14–2.18)	0.0047	0.852 (0.797–0.900)	0.844 (0.791–0.891)	0.0041
Men	248	1.72 (1.16–3.06)	0.023	0.890 (0.823–0.948)	0.876 (0.802–0.939)	0.019
Women	341	1.27 (0.81–2.14)	0.23	0.840 (0.777–0.899)	0.836 (0.768–8951)	0.23
miR-19b-3p						
All	597	1.50 (1.11–2.04)	0.0069	0.854 (0.854–0.903)	0.846 (0.793–0.899)	0.0065
Men	250	1.51 (0.96–3.04)	0.094	0.886 (0.815–0.947)	0.876 (0.797–0.939)	0.086
Women	347	1.44 (0.93–2.59)	0.071	0.845 (0.784–0.904)	0.839 (0.770–0.899)	0.074

Logistic regression analysis assessing the prognostic value of whole blood miRNAs for hypertension diagnosed during the period between 2011 and 2018–2020 YFS follow-ups. AUC_base_ and AUC_miRNA_ represent area under receiver operating characteristics (ROC) curves before and after miRNA inclusion, indicating discrimination. Likelihood ratio test *P*-values (pLR) indicate whether miRNA inclusion significantly improved model fit.

**FIGURE 3:**
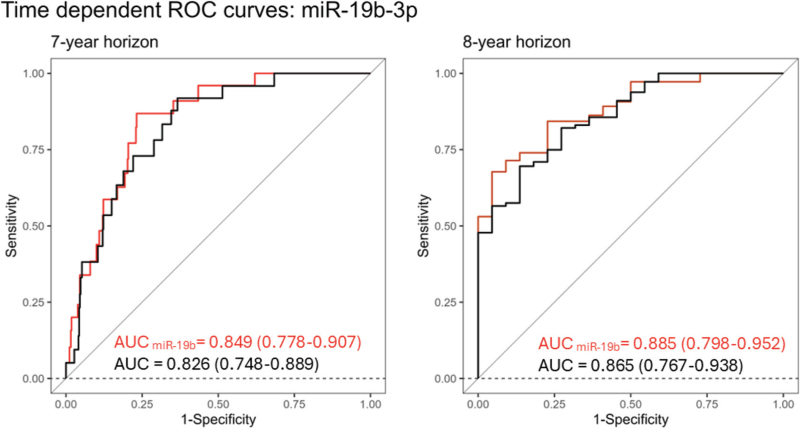
Time-dependent receiver operating characteristics (ROC) curves on improved discrimination after miR-19b-3p addition at 7- and 8-year horizons.

To evaluate time-to-event prediction, we refitted the FHS hypertension risk model [34] to the YFS using Weibull regression. The model included sex, age, SBP and DBP, BMI, smoking, parental hypertension, and interaction between DBP and age. Among the candidate miRNAs, miR-19b-3p remained statistically significant in the Weibull model after multiple testing correction (*P*_FDR_ = 0.0092, Table S10) with a hazard ratio of 1.46 (95% CI 1.15–2.00, *P*_FDR_ = 0.005), indicating an increased risk of hypertension onset with higher miR-19b-5p miRNA levels. Inclusion of miR-19b-3p in the Weibull model significantly improved model fit (LR test, *P* = 0.0012) and enhanced discrimination performance at 7 and 8-year time horizons, as measured by time-dependent c-statistics (Fig. 3). In sex-stratified analysis, miR-19b-3p retained statistical significance for men (*n* = 250), with hazard ratio 2.13 (95% CI 1.38–4.45, *P* = 5.97×10^–5^).

### Identification of correlated miRNA target genes and pathway analysis

We analyzed the correlation between miR-19a-3p, miR-19b-3p, and miR-329–3p levels and their validated target genes expressed in whole blood. Among the validated targets, only a positive correlation between miR-19b-3p and *FYCO1* expression remained significant after FDR correction (*r* = 0.18, *P*_FDR_ = 3.6 × 10^–4^). The detailed findings, including those significant with nominal *P*-values, are summarized in Table S10.

## DISCUSSION

To our knowledge, this is the first study using a large longitudinal cohort to evaluate prospective associations between high-throughput circulatory miRNA profiles and hypertension diagnosis. We assessed the risk prediction potential of circulatory miRNAs that showed cross-sectional association with BP or had a prospective association with hypertension diagnosis in YFS whole blood. We demonstrate that circulatory miRNAs – specifically miR-19a-3p, miR-19b-3p, and miR-329–3p – have a prospective association with hypertension onset.

Regarding cross-sectional findings, in the whole blood miRNomics of the YFS, we identified 15 miRNAs that were significantly (*P*_FDR_ < 0.05) correlated with at least one BP parameter. Of these, nine remained significantly (*P* < 0.05) associated with BP after adjusting for sex, age, and BMI, while seven retained significances after further adjustment for smoking, alcohol usage, ultrasound-identifiable steatotic liver disease, work-related stress, T2D, TC, and BP medication. Most of these associations were also significant in normotensive individuals, suggesting they may have a role in normal BP regulation. Despite differences in blood sample types, three of the BP-associated miRNAs identified in whole blood were also observed in serum, and four in plasma (LURIC).

We did not observe significant (*P*_FDR_ < 0.05) cross-sectional differences in whole blood miRNA levels between individuals with and without an hypertension diagnosis, which contrasts with previous studies that also used unbiased miRNA profiling [17,19,21,25,30,50]. Specifically, the population-based whole blood miRNomics study by Matshazi *et al.*[19] – a study with a similar setting to ours – reported significant differences between individuals diagnosed with HT prior to the study and those without an hypertension diagnosis. Instead, we identified significant differences between individuals with elevated BP and those with normal BP, as defined by BP measurements taken during the study visit, which are discussed later. However, the miRNAs identified in our study were different from those reported by Matshazi *et al.*[19], who also conducted a similar comparison between screen-detected hypertensive and normotensive individuals. This discrepancy may be explained by the stricter multiple testing correction applied in our study, the larger sample size, or the ethnic differences between the study populations (white Europeans vs. ethnically diverse South-Africans).

Our results support previous findings of reduced miR-150–5p and miR-200c-3p in hypertension, as both miRNAs showed negative association with BP parameters [25,51]. These miRNAs are known to influence endothelial cells: miR-200c impairs endothelial function, while miR-150, which is highly abundant in lymphocytes [52], is involved in endothelial lineage specification and vasculogenesis [53]. Inflammatory conditions such as T1D, T2D, and chronic psoriasis lead to elevated miR-200c within the vascular wall, accompanied by a decrease in circulating levels [54–56]. Elevated miR-200c in the vascular wall contributes to endothelial cell apoptosis, oxidative stress, and senescence, as well as lower nitric oxide levels and impaired endothelium-dependent vascular relaxation [55,57]. These mechanisms are likely relevant to hypertension as well, given the role of inflammation and oxidative stress in its pathophysiology [58].

Whole blood associations between BP and miR-122–5p and miR-885–5p were also observed in serum, with higher whole blood levels of miR-122–5p distinguishing hypertensive individuals from normotensives. Both miR-122–5p and miR-885–5p are highly expressed in liver tissue, and their circulatory levels have been previously linked to liver enzyme levels and nonalcoholic fatty liver disease (NAFLD) [17,39]. Given that NAFLD is a common comorbidity of hypertension [59], there is a possibility that the associations between miR-122 and miR-885 with BP could be confounded by liver health. For instance, prior studies linking serum and plasma miR-122 to hypertension did not account for liver health, which may have contributed to the inconsistent direction of association with hypertension reported in these studies [60–62]. Nevertheless, our study found that the independent positive associations between whole blood miR-122–5p and BP, and serum miR-885–5p and BP, remained significant even after adjusting for ultrasound-detected liver steatosis, among other covariates. In addition to liver, these miRNAs are also abundant in peripheral blood mononuclear cells, which might suggest their liver-independent function [63–65].

In addition to miR-122–5p, whole blood miR-144–5p was downregulated in hypertensive individuals as well as negatively associated with both DBP and SBP. The downregulation of miR-144 has been suggested as a general indicator of pathologies and reduced overall health [66]. In a previous study, we also demonstrated that whole blood miR-144–5p is reduced in individuals with impaired fasting glucose and T2D [40]. However, whole blood miR-144–5p negatively associated with both BP parameters and was lower in hypertensive individuals, even after adjusting for T2D, among other factors included in fully adjusted model.

Several whole blood miRNAs associated with BP in our study, including miR-144–5p [67], miR-183–5p [68], miR-185–5p [69–71], and miR-363–5p [72,73], have been mechanistically linked to either atheroprotective or atherogenic roles in *in vitro* or *in vivo*, as well as to CVDs in population-based studies.

Among miRNAs associated with incident hypertension, miR-329–3p also provided additional prognostic value beyond traditional hypertension risk factors and MAP. Its expression was inversely associated with incident hypertension, suggesting a potential protective role against future hypertension. Notably, miR-329–3p showed no cross-sectional association with BP, and its added predictive value on top of MAP suggests it may influence hypertension risk through alternative mechanisms that warrant further investigation. While most research on miR-329–3p is focused on its role in cancer, available in-vitro and in-vivo evidence suggests it may also be involved in angiogenesis and neovascularization [74–76], and metabolic reprogramming associated with vascular remodeling in pulmonary HT [77].

miR-19a-3p and miR-19b-3p were associated with both cross-sectional BP and incident hypertension. miR-19b-3p also associated with time-to-onset of hypertension and improved risk prediction performance at clinically relevant time horizons in survival analysis. These results highlight the potential of miR-19b-3p to identify individuals at risk for hypertension and to predict when the disease is likely to develop. While elevated levels of these miRNAs were linked to future hypertension, they negatively correlated with BP in whole blood and plasma, suggesting potential short-term protective effects against elevated BP. However, in the long-term, their higher levels in healthy individuals were associated with an increased hypertension risk and, in the case of miR-19b-3p, with a more rapid progression to disease onset. The negative cross-sectional association with BP contrasts with a recent study reporting a positive correlation between plasma miR-19a-3p, miR-19b-3p, and BP, particularly in obese individuals. However, the same study also found that weight loss associated reduction in these miRNAs correlated with decreased atherosclerotic CVD risk, supporting the idea that elevated miR-19a-3p and miR-19b-3p may be unfavorable to long-term cardiovascular health [78].

MicroRNAs miR-19a-3p and miR-19b-3p have been linked to both atheroprotective and pro-atherosclerotic roles, with mixed findings regarding their association with CAD [79–85]. Elevated levels of miR-19b-3p have also been observed in acute heart failure patients [86], and in-vivo studies suggest that both miRNAs promote heart regeneration [87] and have protective effects against hypertension-induced cardiac hypertrophy [88]. Given that hypertension often co-occurs with these CVDs, our findings of an independent link between these miRNAs and future hypertension diagnosis in a middle-aged cohort suggest that they may contribute to elevated CVD risk also through their potential role in hypertension.

Our study has a notable advantage over similar research due to its use of a large follow-up cohort and unbiased miRNA profiling. The YFS data provides substantial statistical power to identify novel associations between miRNAs, BP, and future hypertension diagnosis. To our knowledge, this is the first study to evaluate prospective relationships between nonpreselected miRNAs and future hypertension diagnosis as well as their potential as risk biomarkers. There are also limitations in our study. In prospective analysis, modest number events relative to the number of predictors and the use of Weibull survival regression that is based on interval censored data may influence the stability of the estimated coefficients and the model performance merits. BP was measured in a single office visit, which may not capture accurately long-term variations in BP, while hypertension diagnosis relied on self-reported data, which can underestimate true prevalence particularly for individuals with stage 1 hypertension [89,90]. Our findings are also based on miRNA measurement from a single time point, which may not accurately reflect the long-term levels of miRNA in circulation. The heterogeneous cell population of whole blood prevents determination of miRNA origin. Utilizing whole blood mRNA data for the target analyses did not reveal significant associations, likely because the main function of the miRNAs is carried out in other tissues. Lastly, while some cross-sectional findings in whole blood were replicated in YFS 2011 serum replicate and plasma of LURIC participants, cross-sample comparison is constrained by differences in miRNA pool diversity, profiling platforms, and normalization techniques [17,91]. Thus, our cross-sectional and prospective findings are exploratory and require validation in independent cohorts.

To conclude, this exploratory population-based study of middle-aged adults uncovered novel associations between miRNAs, hypertension, and BP, while also replicating previous findings. We identified several miRNAs being associated with BP, even among normotensive individuals, suggesting their potential role in BP regulation. We also identified miRNAs that are dysregulated in individuals with high BP, specifically miR-122–5p and miR-144–5p. Given the significant continuous associations between miRNAs and BP, along with variations in miRNA levels based on BP thresholds rather than hypertension diagnosis, our findings suggest that miRNA levels may reflect ongoing BP levels rather than a fixed disease state. Most importantly, we identified that miR-19a-3p, miR-19b-3p, and miR-329–3p associated with incident hypertension. Specifically, miR-329–3p appeared protective and added risk prediction value beyond traditional risk factors, while miR-19b-3p was also associated with time-to-onset, contributing improved predictive performance at clinically relevant 7 and 8-year time horizons. These findings provide a basis for further research to clarify the role of miRNAs in BP regulation and hypertension development, as well as potential for hypertension risk assessment.

## ACKNOWLEDGEMENTS

None.

D.K. was supported by funding from Aarne Koskelo Foundation, The Finnish Foundation for Cardiovascular Research, and the Tampere City Science Fund. PPM was supported by the Academy of Finland (Grant number: 349708). M.E. was supported by the Academy of Finland (Grant number: 339390)

The Young Finns Study has been financially supported by the Academy of Finland: grants 356405, 322098, 286284, 134309 (Eye), 126925, 121584, 124282, 129378 (Salve), 117797 (Gendi), and 141071 (Skidi); the Social Insurance Institution of Finland; Competitive State Research Financing of the Expert Responsibility area of Kuopio, Tampere and Turku University Hospitals (grant X51001); Juho Vainio Foundation; Paavo Nurmi Foundation; Finnish Foundation for Cardiovascular Research; Finnish Cultural Foundation; The Sigrid Juselius Foundation; Tampere Tuberculosis Foundation; Emil Aaltonen Foundation; Yrjö Jahnsson Foundation; Signe and Ane Gyllenberg Foundation; Diabetes Research Foundation of Finnish Diabetes Association; EU Horizon 2020 (grant 755320 for TAXINOMISIS and grant 848146 for To Aition); European Research Council (grant 742927 for MULTIEPIGEN project); Tampere University Hospital Supporting Foundation, Finnish Society of Clinical Chemistry, the Cancer Foundation Finland, and BETTER4U_EU (Preventing obesity through Biologically and bEhaviorally Tailored inTERventions for you; project number: 101080117).

The genome-wide methylation analyses in the LURIC cohort were financially supported by the German Federal Ministry for Education and Research (BMBF; grant agreement numbers 01EA1808A and 01EA1411A) within the framework of the German Competence Cluster for Nutrition and Cardiovascular Health (nutriCARD) (Halle-Jena-Leipzig) and by the 7th Framework Program RiskyCAD (grant agreement number 305739) of the European Union.

The funders had no role in study design, data collection and analysis, decision to publish, or preparation of the manuscript.

D.K., A.B., and E.R. planned the study. D.K., A.B., and E.R. designed the data analysis and D.K. and A.B. performed the analyses. E.R., L.P.L., and S.M., and instructed in the data analysis, while N.M., L.P.L., I.S., P.P.M., M.J., M.W., M.E., M.K., N.H., O.R., M.E.K., W.M., and T.L. provided data. N.O., M.K., O.R., T.L., and E.R. acquired funding and provided general leadership. D.K. wrote the first draft of the manuscript and edited it in response to the comments of the co-authors. A.B., S.R., J.C., S.M., and E.R. participated in the manuscript writing and all co-authors read and revised the final manuscript.

Supplementary Information: Supplemental Methods [37,39,92–96].

Tables S1-S10.

### Conflicts of interest

The authors declare no conflict of interest.

## Supplementary Material

Supplemental Digital Content

## Supplementary Material

Supplemental Digital Content
