# Supplementary material for: Prognostic potential of circulatory miR-19a-3p, miR-19b-3p, and miR-329–3p for future hypertension diagnosis
Source: J Hypertens. 2026 Mar 5;44(5):811–22. doi: 10.1097/HJH.0000000000004272 (PMC13034753; doi:10.1097/HJH.0000000000004272)
Supplement: Supplemental Digital Content [file jhype-44-811-s001.docx]

# Supplementary file contents

| **Supplementary File 1 (Word)** | *Measurement and definition of covariates* |
| --- | --- |
| **Supplementary File 2 (Excel)** | |
| Supplementary Table 1 | *YFS 2011 follow-up whole blood subset demographics* |
| Supplementary Table 2 | *YFS 2011 follow-up serum subset demographics* |
| Supplementary Table 3 | *YFS 2011 and 2018-2020 follow-up whole blood subset* *demographics* |
| Supplementary Table 4 | *LURIC serum subset demographics* |
| Supplementary Table 5 | *Correlation of circulatory miRNAs with blood pressure* |
| Supplementary Table 6 | *Association analysis of circulatory miRNAs with blood pressure* |
| Supplementary Table 7 | *Association analysis of whole blood miRNAs with blood pressure in normotensives* |
| Supplementary Table 8 | *Sex-stratified association analysis of whole blood miRNAs with blood pressure* |
| Supplementary Table 9 | *Prognostic logistic regression analysis with multiple testing correction* |
| Supplementary Table 10 | *Weibull regression analysis with multiple testing correction* |
| Supplementary Table 11 | *Correlation between prognostic miRNAs with their gene targets expressed in whole blood* |

# Measurements of covariates

## Clinical and biochemical measurements

In Young Finns Study (YFS), venous blood samples were drawn after overnight fast. For the biochemical measurements, serum was separated, aliquoted and stored at -70 °C until analysis. Glucose concentrations were measured with Glucose System Reagent (Beckman Coulter Biomedical) and serum alanine aminotransferase (ALT), aspartate aminotransferase (AST), gamma-glutamyl transferase (GT), glucose, cholesterol, and triglyceride concentrations were measured with ALT, AST, GT, Glucose, Cholesterol, and Triglycerides System Reagent, (Beckman Coulter Biomedical) with an AU400 instrument (AU400, Olympus). Glycated hemoglobin (HbA1c) fraction in whole blood was measured by an Abbott Architect ci8200 analyzer (Abbott Laboratories).The concentration of total hemoglobin was first determined calorimetrically, after which the concentration of HbA1c was measured immunoturbidimetrically using the microparticle agglutination inhibition method (Fisher Diagnostics). These two concentrations were used to calculate the HbA1c percentage (HbA1c%).

In Ludwigshafen Risk and Cardiovascular Health (LURIC), venous blood samples were drawn after overnight fast. For the biochemical measurements serum was separated, aliquoted and snap frozen in liquid nitrogen and stored at -80 °C until analysis. Serum alanine aminotransferase (ALT), aspartate aminotransferase (AST), gamma-glutamyl transferase (GT), glucose, cholesterol and triglycerides were measured enzymatically with ALT, AST, GT, GLU, CHOL and TG reagents Hitachi 717 analyzer (Hitachi). Details on biochemical measurements for YFS and LURIC are provided in Raitoharju et al., 2016 and Winkelmann et al., 2001, respectively.

## Type 2 diabetes

In YFS, diagnosis of type 2 diabetes (T2D) included subjects with a fasting plasma glucose level of over 7.0 mmol/l or HbA1c of over 6.5 % (48 mmol/mol), or those with reported use of oral glucose-lowering medication or insulin (but had not reported having type 1 diabetes) or who had a reported diagnosis of T2D by a physician. In LURIC, T2D was diagnosed with a fasting plasma glucose over 7.0 mmol/l or a 2h plasma glucose concentration over 11.0 mg/dl after an oral glucose test, or a history of T2D (self-reported diagnosis or antidiabetic medication) (Raitoharju et al., 2016; Winkelmann et al., 2001).

## Liver ultrasound

Liver ultrasonography was performed to assess liver steatosis. Examinations were done by a trained sonographer using Acuson Sequoia 512 ultrasound mainframes with validated protocols (Edens et al., 2009; Saverymuttu et al., 1986). YFS participants were then classified in two groups based on ultrasound-detectable steatotic liver disease (present/absent) (Raitoharju et al., 2016). Given the absence of liver steatosis assessments via ultrasound in LURIC, ALT levels were utilized as an alternative liver health marker in regression analyses.

## Alcohol consumption, smoking, work-related stress and parental hypertension

YFS and LURIC participants completed questionnaires about smoking habits and alcohol consumption. In YFS participants reported alcohol intake in the previous week, which was then calculated into daily units of alcohol (8 g pure alcohol/day). For statistical analyses, alcohol consumption was categorized into four groups (no alcohol intake during the last week; 2) >0 to <2 units of alcohol; 3) 2 to <4 units of alcohol per day; 4) >= 4 units of alcohol per day) (Suomela et al., 2016). Regarding smoking, YFS participants were classified into either daily smokers or non-smokers based on their responses.

In LURIC, participants reported their frequency of beer, wine and spirits consumption. The reported portions were converted into volume: 300 ml for beer, 100 ml for wine, and 30 ml for spirits. The average alcohol contents (5% for beer, 12% for wine, and 43% for spirits) were used to calculate pure alcohol consumption by multiplying the volume by the alcohol percentage and the weight of one milliliter of alcohol (0.8 g/ml). (Moissl et al., 2021).

In the 2007 YFS follow-up, participants completed questionnaires regarding their parent morbidities, and work-related stress (Occupational Stress Questionnaire). Based on their responses. participants were categorized according to parental hypertension status (none, one, or both parents affected). Work-related stress, defined as the combination of high demands and low control, was calculated, and participants were divided into high stress and low stress groups. The methodology for measuring work-related stress in YFS was previously reported in more detail (Törnroos et al., 2013).

# References:

Edens, M. A., Van Ooijen, P. M. A., Post, W. J., Haagmans, M. J. F., Kristanto, W., Sijens, P. E., Van Der Jagt, E. J., & Stolk, R. P. (2009). Ultrasonography to Quantify Hepatic Fat Content: Validation by ^1^ H Magnetic Resonance Spectroscopy. *Obesity*, *17*(12), 2239–2244. https://doi.org/10.1038/oby.2009.154

Moissl, A. P., Delgado, G. E., Krämer, B. K., Dawczynski, C., Stojakovic, T., März, W., Kleber, M. E., & Lorkowski, S. (2021). Alcohol consumption and mortality: The Ludwigshafen Risk and Cardiovascular Health (LURIC) study. *Atherosclerosis*, *335*, 119–125. https://doi.org/10.1016/j.atherosclerosis.2021.08.014

Raitoharju, E., Seppälä, I., Lyytikäinen, L.-P., Viikari, J., Ala-Korpela, M., Soininen, P., Kangas, A. J., Waldenberger, M., Klopp, N., Illig, T., Leiviskä, J., Loo, B.-M., Oksala, N., Kähönen, M., Hutri-Kähönen, N., Laaksonen, R., Raitakari, O., & Lehtimäki, T. (2016). Blood hsa-miR-122-5p and hsa-miR-885-5p levels associate with fatty liver and related lipoprotein metabolism—The Young Finns Study. *Scientific Reports*, *6*(1), 38262. https://doi.org/10.1038/srep38262

Saverymuttu, S. H., Joseph, A. E., & Maxwell, J. D. (1986). Ultrasound scanning in the detection of hepatic fibrosis and steatosis. *BMJ*, *292*(6512), 13–15. https://doi.org/10.1136/bmj.292.6512.13

Suomela, E., Oikonen, M., Pitkänen, N., Ahola-Olli, A., Virtanen, J., Parkkola, R., Jokinen, E., Laitinen, T., Hutri-Kähönen, N., Kähönen, M., Lehtimäki, T., Taittonen, L., Tossavainen, P., Jula, A., Loo, B.-M., Mikkilä, V., Telama, R., Viikari, J. S. A., Juonala, M., & Raitakari, O. T. (2016). Childhood predictors of adult fatty liver. The Cardiovascular Risk in Young Finns Study. *Journal of Hepatology*, *65*(4), 784–790. https://doi.org/10.1016/j.jhep.2016.05.020

Törnroos, M., Hintsanen, M., Hintsa, T., Jokela, M., Pulkki-Råback, L., Hutri-Kähönen, N., & Keltikangas-Järvinen, L. (2013). Associations between Five-Factor Model traits and perceived job strain: A population-based study. *Journal of Occupational Health Psychology*, *18*(4), 492–500. https://doi.org/10.1037/a0033987

Winkelmann, B. R., März, W., Boehm, B. O., Zotz, R., Hager, J., Hellstern, P., & Senges, J. (2001). Rationale and design of the LURIC study—A resource for functional genomics, pharmacogenomics and long-term prognosis of cardiovascular disease. *Pharmacogenomics*, *2*(1s1), S1–S73. https://doi.org/10.1517/14622416.2.1.S1
